# Cost of delivering health care services at primary health facilities in Ghana

**DOI:** 10.1186/s12913-017-2676-3

**Published:** 2017-11-17

**Authors:** Maxwell Ayindenaba Dalaba, Paul Welaga, Chieko Matsubara

**Affiliations:** 1 Navrongo Health Research Centre, Box 114, Navrongo, Ghana; 2 Bureau of International Medical Cooperation, National Center for Global Health and Medicine, Tokyo, Japan

**Keywords:** Cost, Primary health care, Health centre, Community-based Health Planning and Services, Upper West Region, Ghana

## Abstract

**Background:**

There is limited knowledge on the cost of delivering health services at primary health care facilities in Ghana which is posing a challenge in resource allocations. This study therefore estimated the cost of providing health care in primary health care facilities such as Health Centres (HCs) and Community-based Health Planning and Services (CHPS) in Ghana.

**Methods:**

The study was cross-sectional and quantitative data was collected from the health provider perspective. Data was collected between July and August, 2016 at nine primary health facilities (six CHPS and three HCs) from the Upper West region of Ghana. All health related costs for the year 2015 and revenue generated for the period were collected. Data were captured and analysed using Microsoft excel. Costs of delivery health services were estimated. In addition, unit costs such as cost per Outpatient Department (OPD) attendance were estimated.

**Results:**

The average annual cost of delivering health services through CHPS and HCs was US$10,923 and US$44,638 respectively. Personnel cost accounted for the largest proportion of cost (61% for CHPS and 59% for HC). The cost per OPD attendance was higher at CHPS (US$8.79) than at HCs (US$5.16). The average Internally Generated Funds (IGF) recorded for the period at CHPS and HCs were US$2327 and US$ 15,795 respectively. At all the facilities, IGFs were greatly lower than costs of running the health facilities. Also, at both the CHPS and HCs, the National Health Insurance Scheme (NHIS) reimbursement was the main source of revenue accounting for over 90% total IGF.

**Conclusions:**

The average annual cost of delivering primary health services through CHPS and HCs is US$10,923 and US$44,638 respectively and personnel cost accounts for the major cost. The government should be guided by these findings in their financial planning, decision making and resource allocation in order to improve primary health care in the country. However, more similar studies involving large numbers of primary health facilities in different parts of the country are needed to assess the cost of providing primary health care.

**Electronic supplementary material:**

The online version of this article (10.1186/s12913-017-2676-3) contains supplementary material, which is available to authorized users.

## Background

Ghana like many other Africa countries is faced with preventable and treatable diseases such as malaria, maternal and child deaths, diarrhea, malnutrition, HIV/AIDS and other diseases. The most affected people are those living in remote areas where they find it very difficult to access appropriate health care. In the light of this, the primary health care is being strongly advocated to bridge the equity gap in geographical access to quality health care.

Primary health care (PHC) is operationally defined as “essential care based on practical, scientifically sound and socially acceptable methods and technology, made universally accessible to individuals and families in the community through their full participation, and at a cost that the community and country can afford to maintain at every stage of their development in the spirit of self-reliance and self-determination” [1]. Primary health care is a paramount approach to achieving Universal Health Coverage(UHC) [2, 3]. According to World Health Report, UHC is defined as all people having full access to needed promotive, preventive, curative, rehabilitative and palliative health care services which should be of a standard quality without having to suffer any financial hardship or impoverishment [4]. The government of Ghana in recent times has prioritized the expansion and strengthening of primary health care such as Health Centres (HCs) and Community-based Health Planning and Services (CHPS) [5] in order to achieve health targets and UHC.

CHPS is a “strategy for health care delivery where cost-effective and adequate basic quality primary health services are provided to individuals and households in the communities where they live through engaging the community in the planning and delivery of services” [5]. CHPS is the most peripheral unit of health care delivery and it provides mainly preventive care with some curative care services for minor illness. Health Centres on the other hand provide basic curative care, disease prevention, maternal and child health services [7]. Referral cases from the primary healthcare level are sent to district and regional hospitals or teaching hospitals where specialized clinical and diagnostic cares are rendered.

Even though primary health care is seen as a strategy for reducing inequities and promoting geographical access to basic health care, it is faced with challenges such as inadequate financing. How much is needed to efficiently run a primary health care facility is not well known because there are limited studies on the cost of running health facilities in Ghana. The few studies conducted in Ghana on health facility costing concentrated on cost of delivering health services at the HCs and hospital level [8, 9]. There has not been any published study on the cost of providing health care at CHPS.

In general, cost efficiency assessment, budgeting and cost effectiveness analysis of health facilities depend largely on the availability of cost information. Heath facility cost information will provide the required information to researchers, policy makers and health care managers in the financial management, planning, budgetary allocations, management of IGF and reduction in health facility inefficiency.

This study therefore sought to fill this crucial gap by examining the costs associated with running primary health facilities such as HCs and CHPS. The study took the health provider perspective and did not include direct patient costs.

### Ghana health care system

In Ghana, health care is mostly provided by the government and generally administered by the Ministry of Health (MOH) and the Ghana Health Service (GHS). The MOH coordinates the implementation of primary health care policy with other ministerial agencies whiles the GHS is the implementing arm of the MOH that is responsible for health service delivery in the country.

The Ghana Health Service consists of government hospitals and clinics, hospitals and clinics within the Christian Health Association of Ghana, and private facilities [9]. Health care services are delivered at three main levels: primary, secondary, and tertiary levels. The primary level is made of all health institutions based at the district level such as Community-based Health Planning and Services (CHPS), clinics, health centres, and hospitals. The secondary level comprised institutions that are based at the regional level such as the regional hospitals whiles the tertiary level comprise the tertiary hospitals such as the teaching hospitals linked to universities that take referrals for advanced and very specialized care [10].

To improve health care and ensure universal access to basic healthcare services to all residents of Ghana, in 2003, the government of Ghana established the National Health Insurance Scheme (NHIS). The NHIS allows people to make contributions into a fund so that in the event of illness contributors could be supported by the fund to receive affordable health care in the health facilities. The NHIS accredits both public and private health facilities in the country to provide medical care at the point of service for those enrolled into the scheme [11]. Those who are enrolled into the scheme use their cards to access care whiles those who are not enrolled pay Out of Pocket (OOP). The NHIS pays for hospitalization and outpatient visits, basic laboratory testing and certain medications for its clients. Enrollment into the NHIS is not encouraging given that after a decade of implementation of the NHIS, only 34% of the national population is currently enrolled into the NHIS [12].

## Methods

### Study area

The study was conducted in the Upper West Region of Ghana (UWR). UWR is one of the 10 Regions in Ghana, located in the northern part of Ghana. Its population is about 680,000 and it is one of the regions with a low population density in Ghana. The Region is bordered by Burkina Faso to its North and West. Subsistence agriculture is the mainstay of the people. The region is in one of the poorest regions in Ghana. The major ethnic groups in the region are the Dagaati, Sissala, and Wala [13].

There are a total of 242 health facilities providing various types of services in the Upper West Region. These are three district government hospitals, one Regional hospital, two Christian Health Association of Ghana hospitals and three private hospitals. The rest are five Polyclinics, 66 health centres, ten clinics and 147 CHPS and four maternity homes [14].

### Selected health facilities

**Fig. 1:**
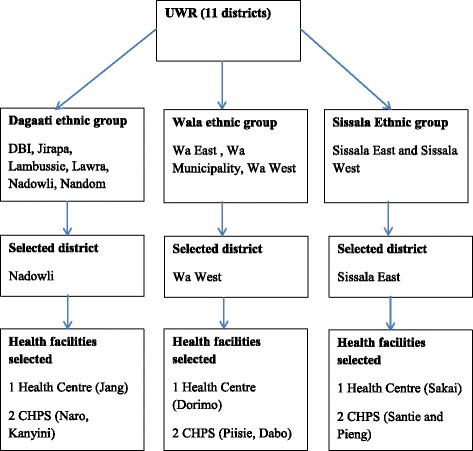
Sampling strategy

A total of nine primary health facilities were selected for the data collection (6 CHPS and 3 HCs). A multi stage sampling method was used for the selection (Fig. 1) of the districts and the health facilities for this study.

We first grouped all the 11 districts in the Upper West region by the three main ethnic groups: Daagati, Wala, and Sissala. The Dagaati districts are made up of Daffiama Bussie Issa (DBI), Jirapa, Lambussie, Lawra, Nadowli, Nandom; Wala districts are made up of Wa East, Wa Municipality, Wa West; and the Sissala districts comprised of Sissala East and Sissala West.

Secondly, we randomly selected one district from each of the three main ethnic groups of districts. Nadowli, Wa West, and Sissala East district were respectively selected to represent Daagati, Wala, Sissala ethnic group in this order. In stage three, we randomly selected one HC and two CHPS from each of the selected districts. Wa West has 7 HCs and 20 CHPS; Sissala East district has 6 HCs and 13 CHPS; whiles Nadowli district has 10 HCs and 17 CHPS [13]. Overall, the three HCs selected were Dorimo, Jang and Sakai and the six CHPS selected were Piisie, Dabo, Naro, Kanyini, Santie and Pieng.

The health facilities selected for this study are typical of the country’s primary health care facilities in terms of infrastructure, equipment, staffing, population coverage and mode of operation. Per the guidelines, primary health care facilities are not supposed to provide inpatient care, there are required to provide only outpatient care. In general, CHPS usually covers a population of 3000 to 4500 people [3] and it is being staffed by at least a trained Community Health Officer (CHO) to provide basic primary health care services such as outpatient and outreach services. CHPS that have qualified personnel (midwife) can conduct deliveries under the midwife’s care [14, 15]. Nevertheless, in a CHPS facility without a midwife, health workers can conduct emergency deliveries in circumstances in which a woman is unable to reach a higher-level health facility in time for delivery [15].

Health Centres on the other hand usually serve a community with a population of 15,000–30,000 people and are supposed to provide outpatient and normal child delivery services [15]. HCs are generally headed by a medical or physician assistant and staffed with program heads in the areas of midwifery, laboratory services, public health, environment and nutrition [15].

### Study design and data collection

The study was cross-sectional and quantitative data was collected from the health provider perspective. Data was collected during July to August, 2016 by the study investigators who were qualified up to PhD level. Data on both capital and recurrent resources spent for the delivery of health services during the last 1 year (January 2015 to 31st December 2015) were collected using a structured questionnaire. One complete year data were collected to cater for seasonal variation of diseases and utilization. Data were collected through document reviews, interviews and physical inventory of resources used in the health facilities within the period. The cost data collected included: personnel, administrative cost/overheads, medicine, consumables, equipment and vehicles. Though efforts were made to obtain cost of building, we were not able to obtain reliable cost of building the health facilities, hence cost of building has not been included in the cost analysis.

#### Personnel cost

This included gross income of all the personnel who worked in the health facility during the reference year.

#### Administrative cost

This cost component included cost on cleaning products, repairs, fuel, lubricants, printing, photocopying, stationary etc.

#### Medicine and consumable costs

Stock registers were reviewed and the quantity of various medicines and medical consumables consumed within the period were obtained. Some unit prices of medicines and consumables could not be obtained from the registers and had to be obtained from the Upper West Regional price list of medicines and non-medicines for 2015 [16].

#### Equipment cost

This included cost of general equipment in the various rooms (waiting room, consulting room etc.) such as fetoscope, thermometer, weighing scales, benches, tables, chairs etc. Though some of the health facilities had solar panels, we were not able to obtain a reliable cost of purchase hence solar panel cost was not included in the cost analysis. Some unit prices of equipment were obtained from the Upper West Regional price list medicines and non-medicines for 2015 [16]. In some cases, unit prices of locally obtained equipment such a benches, tables, chairs etc. were obtained from market sources.

#### Vehicle costs

This included cost of all functional means of transport such as cars and motorbikes in the health facilities that are used for supervision, outreach, home visit and administrative activities. Vehicle cost was basically cost of motorbikes because none of the study facilities had a car or any other form of means of transport.

### Staff time

All the staff in each health facility were interviewed with a structured questionnaire on time allocation for different services in the last 1 week. This included time spent providing services either at the health facilities or as outreach services. At both the HCs and CHPS, all services/ activities were reported to be carried out throughout the week, particularly from Monday-Friday (they were all non-specialized clinic sessions). During the analysis, percentage times spent on activities were then categories into four groups:

1. Curative services: This comprised staff time spent on general consultation, nursing, delivery, laboratory and dispensing drugs.
2. Preventive services: This included staff time spent in conducting Antenatal Care (ANC), vaccination, family planning and Postnatal Care (PNC).Some child vaccinations are sometimes done during expanded programme of immunization (EPI) days. Since EPI is not a regular activity, staff time spent on EPI was not included.
3. Other: This comprised staff time spent in documentation, cleaning etc.

In order to determine the financial sustainability of the health facilities, information on revenue generated for the period at the health facilities were collected through review of records and interviews of key staff. Also to determine health care utilization, OPD register books/consultation books as well as routine reports were reviewed and the number of services provided during the reference period was collected.

### Data analysis

Annual costs were calculated for each of the nine health facilities using a full costing approach. Full cost approach means calculation of both capital and recurrent costs. The total annual costs were estimated using unit prices and quantities consumed within the period. In addition, donated items were valued and included in the cost calculation. All cost were collected in local currency which is Ghana cedis (GH¢) and results presented in US$ using 2015 average interbank exchange rate of 4 Ghana Cedis to 1 US$. Totals, averages and unit costs were estimated.

The cost components included in the analysis were: personnel cost, administrative cost, medicine and consumables cost (representing recurrent cost), cost of equipment and vehicles (representing capital cost). Recurrent costs are items that are used up during a year and are usually purchased regularly. Capital costs are items with lifespan greater than 1 year and are therefore incurred only every few years rather than annually [17, 18].

Capital costs were annualized to allow for differential timing of capital costs. Thus it gives the equivalent annual cost of the capital cost by spreading the capital costs over their useful life years [17]. In this study, capital costs were annualized using a discount rate with their respective useful life years. A discount rate of 3% was chosen, which is usually the rate used in most economic evaluation studies conducted in developing countries [17–21]. Based on expert opinion and literature review a useful life of 5 years was used for equipment and vehicles [17, 19, 21–23].

Total cost for running each health facility in a year was calculated by adding the annual cost on personnel, administrative, medicine, consumables, equipment and vehicles. The average cost of running a health facility was obtained by summing the cost of running all the health facilities divided by the number of health facilities.

#### Cost of curative and preventive services

We used the step-down allocation (SDA) approach as discussed in the WHO manual [19] and Conteh [25] and used in other studies [7, 21, 24, 26, 27] to calculate cost of curative and preventive services. The SDA method identifies the range of resources used to run a facility and then allots the resources step-by step to selected cost centres or department on an allocation basis [17, 25]. Using SDA approach, we followed five main steps: (1) defining the final product, (2) defining cost centres, (3) identifying the full cost for each input, (4) assigning inputs to cost centres, and (5) allocating all costs to final cost centres. Given the structure and operations of the health facilities, all allocations to cost centres were based on the percentage of staff time on each cost center.

#### Unit costs

We calculated cost per OPD (total cost divided by total OPD attendance), cost per capita (total cost divided by total population covered), IGF per OPD visit (total IGF divided by total OPD attendance), IGF per cost (total IGF divided by total cost) were estimated.

Given that health Centres and CHPS are in different levels of care with different resources and mode of operations, analysis and results for HCs and CHPS were conducted and presented separately. Thus we did not combine data on HCs and CHPS to obtain overall aggregated figures.

### Sensitivity analysis

One-way sensitivity analysis was carried out to explore the effect of changes of some variables that are susceptible to change over time or in different settings in order to aid in the generalization of the study results [17]. Given that several studies use 3–5% discount rates, sensitivity analysis was done to assess the effect of using 5% discount rate. The life span of equipment and vehicle was also varied from 5 years to 10 years.

Over the past few years, the government of Ghana has instituted some interventions and policies aimed at increasing the number of nurses at the primary health level. The numbers of nurses at health facilities are expected to increase in the near future. Therefore a sensitivity analysis was conducted using an increase in personnel cost. We tested for a 10% increase in personnel cost.

### Ethical consideration

Ethical approval was obtained from the Navrongo Health Research Centre Institutional Review Board (Approval ID: NHRCIRB232) and the National Center for Global Health and Medicine (NCGM), Japan (Approval ID: NCGM-G-0020510-00). Permission was also sought from the regional health directorate of the Upper West Region, district health directorate of Wa West, Nadowli and Sissala East as well as the heads of the health facilities included in the study.

## Results

### Background characteristics of health facilities

Table 1 presents the background characteristics of the health facilities included in the study. All the CHPS have similar structure having about 6 rooms and the average number of staff per CHPS facility was 3 (median = 2; range = 2–5 staff). Only one CHPS facility (Kanyini CHPS) had more than 2 staff, and the explanation given was that the population they covered was large. At the health Centres, the average number of staff was 7 (median = 9; range = 7–9 staff).

**Table 1:** Background characteristics of health facilities

Health facilities	Staff	Population covered	OPD (all cases and all ages)	Under 5 (all cases)	Percentage of under 5 cases (%)
CHPS					
Piisie	2	2074	563	267	47
Dabo	2	2312	1569	896	57
Kanyini	5	2375	883	345	39
Naro	2	2170	2185	512	23
Peing	2	1736	789	571	72
Sentie	2	2074	1470	604	41
Total	15	12,741	7459	3195	43
Average	3	2124	1243	533	43
Median	2	2122	1177	542	46
Health centre (HC)					
Dorimo	9	19,211	15,432	8575	56
Jang	7	11,225	6244	5340	86
Sakai	9	7702	4262	358	8
Total	25	38,138	25,938	14,273	55
Average	8	12,713	8646	4758	55
Median	9	11,225	6244	5340	56

The average population covered by a CHPS and Health Centre (HCs) were 2124 (range: 1736–2312 and 11,418 (range: 7702–19,211) respectively. Although there were variations in the population covered by the health facilities, the results however showed that all the health facilities did not covered more than the maximum number as stipulated in the guidelines (i.e. maximum of 3000 people for CHPS and 30,000 for HCs).

Both HCs and CHPS facilities are required to only provide Out Patient Department (OPD) services. Results showed that there were wide variations in OPD attendance recorded at both the health centres and CHPS. The average OPD attendance at the CHPS was 1243(median = 1177) and a wide range of between 563 and 2185 OPD visits. Similarly, the average OPD attendance at the HCs was 8646 (median = 6244) with a range between 4262 and 15, 432 visits.

For the health centres, a possible reason for wide variations in OPD attendance could be due to the variations in population they covered. For instance, Dorimo HC had the highest population coverage (19,211) and recorded the highest number of OPD attendance (15,432). However, the trend was not consistent at the CHPS facilities because some CHPS with high population coverage recorded low OPD attendance. The low OPD attendance recorded by CHPS with high population coverage could be due to poor accessibility and poor record keeping. In general, the average number of OPD attendance at HCs was about 7 times higher than the number of OPD attendance at CHPS.

At the health centres, more than 50% of the OPD cases were children under 5 years old (mean cases = 4758; median = 5340) whiles at the CHPS 43% of cases were children under 5 years (mean cases = 533; median cases = 542).

### Staff time on activities at health facilities

Table 2 present staff reported time on various activities in the reference period. At the CHPS facilities, staff reported spending more time on preventive services than the other services. For instance, the average percentage of time spent by staff on preventive services was 51% (median = 50%; range = 43%- 63%) and 34% (median = 33%; range = 30–40%) of staff time was spent on curative services. The very high average time spent on preventive services reported at the Satie CHPS facility (63%) compared to the other CHPS could be due to the fact that one of the staff at the Satie CHPS was a health promotion officer and therefore spent more time on preventive services. At the health centres, more staff time was spent on curative services (52%) than preventive services (32%).

**Table 2:** Percentage of staff time on activities at health facilities (%)

Health facility	Curative (%)	Preventive (%)	Others (%)
CHPS			
Piisie	40	43	17
Dabo	30	50	20
Kanyini	40	52	8
Naro	35	45	20
Peing	30	50	20
Sentie	30	63	7
Average	34	51	15
Median	33	50	19
Health Centre (HC)			
Dorimo	47	43	11
Jang	58	26	16
Sakai	52	28	20
Average	52	32	16
Median	52	28	16

### Cost distribution with capital costs being annualized

The cost components comprised of recurrent costs such as personnel, administration, medicine and consumables; capital costs such as equipment and vehicle costs (Table 3). The average annual costs for providing health services at CHPS and HCs were US$10,922.57 (range: US$1181.85 – US$15,838.58) and US$44,637.78(range: US$28,281.09 –US$67,595.16) respectively. There were little cost variations across the CHPS facilities but a wide cost variations across the HCs. Personnel cost alone accounted for 75% (US$8173.50) and 64% (US$28,623.37) of total cost at CHPS and HCs respectively. The proportion of cost of medicine and consumables was 15% (US$1585.49) at CHPS and 25% (US$11,245.10) at HC.

**Table 3:** Cost distribution (US$)

Health facilities	Personnel	Administration	Medicine and consumables	Equipment	Vehicle	Total
CHPS						
Piisie	5892	560	879	523	328	8182
Dabo	7770	572	1237	381	655	10,614
Kanyini	13,510	939	561	500	328	15,839
Naro	8400	548	3390	198	164	12,700
Peing	6734	248	2606	110	328	10,025
Sentie	6734	244	840	140	218	8176
Total	49,041	3110	9513	1852	2020	65,535
Average	8174	518	1585	309	337	10,923
Median	7252	554	1058	290	328	10,319
Percent of total cost (%)	75	5	15	3	3	100
Health Centre (HC)						
Dorimo HC	39,564	2201	17,348	2915	5568	67,595
Jang HC	24,131	722	11,786	1234	164	38,037
Sakai HC	22,175	649	4602	637	218	28,281
Total	85,870	3572	33,735	4786	5950	133,913
Average	28,623	1191	11,245	1595	1983	44,638
Median	24,131	722	11,786	1234	218	38,037
Percentage of total cost (%)	64	3	25	4	4	100

By disaggregating cost into capital and recurrent costs, recurrent costs accounted for the largest proportion of total cost. Thus at CHPS, recurrent costs accounted for about 94% of total, whiles at the HCs, recurrent cost was 85% of total cost. The cost of providing health services at HCs for a year was 4 times higher than at CHPS.

### Cost attributed to curative and preventive services

Total cost attributed to curative, preventive services and other services were estimated. At the CHPS level, the average cost attributed to curative services was US$3672 (median = US$3619; range = US$1865- US$5598), average cost for preventive services was US$ 6071 (median = US$5846; range = US$4778 – US$7957) and cost attributable to other services was US$ 1179 (median = US$1002; range = US$419 – US$2285). Preventive services accounted for about 56% of total cost, whiles curative services accounted for 34% of total cost.

At the health centre level, the average cost attributed to curative services, preventive services and other services were estimated at US$24,984(median = US$20,767; range = US$13,106 – US$41,079), US$12,625(median = US$ 13,261; range = US$10,644 – US$13,969), US$7028.82 (median = US$6626; range = 28,281–67,595) respectively. Curative services accounted for about 56% of total costs, whiles preventive services accounted for about 28% of total cost (Table 4).

**Table 4:** Cost attributed to curative and preventive services (US$)

Health Facility	Curative	Preventive	Other	Total
CHPS				
Piisie	2389	4778	1016	8182
Dabo	3850	5800	964	10,614
Kanyini	5598	7957	2285	15,839
Naro	4942	6354	1404	12,700
Puing	3389	5648	989	10,025
Santie	1865	5892	419	8176
Total	22,032	36,428	7075	65,535
Average	3672	6071	1179	10,923
Median	3619	5846	1002	10,319
Percentage of total cost (%)	34	56	11	100
Health Centre(HC)				
Dorimo HC	41,079	13,261	13,255	67,595
Jang HC	20,767	10,644	6625	38,037
Sakai HC	13,106	13,969	1206	28,281
Total	74,953	37,874	21,086	133,913
Average	24,984	12,625	7029	44,638
Median	20,767	13,261	6625	38,037
Percentage of total cost (%)	56	28	16	100

### Sensitivity analysis

The results from the sensitivity analysis showed that varying the discount rate applied to capital costs such as equipment and vehicles from 3% to 5% and life expectancy of from 5 years to 10 years has very little impact on annual costs. A sensitivity was also conducted varying personnel cost. For instance, given that we expect the number of staff to increase in future due to government policies to improve health care, if there is a 10% increase in personnel cost at a CHPS, the average annual cost of providing care will increase by 14% (from US$10,923 to US$11,740) .

### Revenue recorded at the health facilities

Table 5 presents revenue accrued in 2015 in the various health facilities. Two health facilities could not provide revenues accrued within the period because they don’t have records on that. The revenues generated during the period were internally generated funds (IGF) either through out-of-pocket (OOP) payments or through national health insurance reimbursement from insured clients.

**Table 5:** Revenue recorded at the health facilities (US$)

Health facilities	Internally generated funds (out-of-pocket payments)	Internally generated funds (NHIS reimbursement)	Total revenue	Total cost	Revenue as a percentage of cost (%)
CHPS					
Piisie	74	1307	1380	8182	17
Dabo	275	4123	4398	10,614	41
Kanyini	Missing data	1403	1403	15,839	9
Naro	653	1746	2399	12,700	19
Peing	54	2146	2200	10,025	22
Sentie	186	1993	2179	8176	27
Total	1242	12,718	13,960	65,535	21
Average	207	2120	2327	10,923	21
Median	130	1869	2189	10,319	21
Percentage of total cost (%)	9	91	100	100	100
Health centre (HC)					
Dorimo HC	2250	21,250	23,500	67,595	35
Jang HC	Missing data	16,871	16,871	38,037	44
Sakai HC	449	6564	7013	28,281	25
Total	2699	44,685	47,384	133,913	35
Average	900	14,895	15,795	44,638	35
Median	449	16,871	16,871	38,037	44
Percentage of total cost (%)	6	94	100	100	100

The results of this study revealed that, at CHPS, an average of US$2327 was recorded as IGF (median = 2189; range = 1380–4398). Out of this amount, about 91% (average amount = US$2120) was from national health insurance reimbursement and about 9% (average amount = US$ 207) from out-of-pocket (OOP) payments.

At the health centres, the average revenue generated within the period was US$15,795 (median = 38,037; range = 7013–23,500). Out of this amount, about 94% (US$14,895) was from national health insurance reimbursement and about 6% (US$ 899.63) from OOP payments. Clearly, in all the health facilities, the cost of running health facilities exceeded the revenue generated. The average revenue generated as a percentage of total health facility cost was 21% (range = 7% – 41%).

### Unit costs

Table 6 shows unit costs on some key variables by health facility. The cost per OPD attendance at the CHPS was higher than at the HC. For instance, the average health facility cost per OPD attendance at CHPS was US$8.79 (median = US$8.79) with a range of US$5.56 to US$14.53, whereas the average health facility cost per OPD attendance at HC was US$5.16 (median = US$6.09) with range of US$4.38 to US$6.64. Similarly, the health facility cost per capita was higher at CHPS than HC. The health facility cost per capita per year at CHPS and HC were US$5.14(median = US$5.18; range = US$ 3.94 – US$6.67) and US$3.51(median = US$3.52; range = US$3.52 – US$3.67) respectively. The average IGF per OPD attendance was US$1.87(median = US$2.02; range = US$1.10–US$2.80) at CHPS level and US$1.83 (median = US$1.65; range: US$1.52 US$2.70) at HC level. The average IGF per health facility cost was US$0.21 (median = US$0.21; range = US$0.09 – US$0.41) at CHPS level and US$0.35 (median = US$0.35; range; US$0.25 – US$0.44) at HC level.

**Table 6:** Unit costs (US$)

	Cost per OPD	Cost per capita	IGF per OPD	IGF per Cost
CHPS				
Piisie	14.53	3.94	2.45	0.17
Dabo	6.76	4.59	2.80	0.41
Kanyini	17.94	6.67	1.59	0.09
Naro	5.81	5.85	1.10	0.19
Peing	12.71	5.77	2.79	0.22
Sentie	5.56	3.94	1.48	0.27
Total	8.79	5.14	1.87	0.21
Average	8.79	5.14	1.87	0.21
Median	9.74	5.18	2.02	0.21
Health Centre (HC)				
Dorimo HC	4.38	3.52	1.52	0.35
Jang HC	6.09	3.39	2.70	0.44
Sakai HC	6.64	3.67	1.65	0.25
Total	5.16	3.51	1.83	0.35
Average	5.16	3.51	1.83	0.35
Median	6.09	3.52	1.65	0.35

## Discussion

This study analyzed the annual cost of providing services at HCs and CHPS using full costing approached were both capital and recurrent costs were included in the analysis. The study result showed that there are wide variations in costs across the HCs but minimal cost variations across CHPS. A possible explanation for the variations in cost at the CHPS could be due to differences in the number of staff (personnel cost). The variations in HCs could be due to the differences in populations they cover and the total number of people they attended to within the period (differences in facility utilization).

The average annualized cost of delivering services through a CHPS for a year was US$10,923 and that for a HC was US$44,638. Recurrent cost accounted for about 80% of total costs in both the HCs and CHPS. The annual cost of delivering services at a HC in our study (US$44,637.78) was lower than what has been estimated in other studies conducted in Ghana. For instance, in the study conducted in Kassena-Nankana district of northern Ghana, the cost of running a health centre for a year was estimated at US$136,014. Nevertheless, the high cost difference could be due to the fact that our study did not include cost of building in the analysis and also due to difference in period of data collection.

Personnel cost accounted for the largest proportion of annualized cost (61% for CHPS and 59% for HC). The results from the sensitivity analysis also showed that an increase in personal cost through increase in the number of staff has great impact on annual costs. Our results is consistent with other studies where personnel cost emerged as the highest cost component [6, 7, 26, 28, 29]. Personnel cost is an important cost variable in the provision of primary health care which policy makers should consider when planning on the expansion of primary health care. In Ghana, payment of salaries of government workers is one of the greatest burdens that the government is grappling with in recent times. The constant agitations by health workers for salary increases coupled with the single spine salary scale is greatly affecting government funds for service provision. For instance, the government spent 58% of its full-year budget for state-worker pay in the first 6 months of 2016. Payment of government workers salaries usually account for more than 50% of the national health sector budget allocations [30].

Much of the staff time at the CHPS facilities was spent on preventive services (51%). In addition, the largest proportion of cost was spent on preventive care (56% of total cost). This is in line with the CHPS concept that places much importance on preventive health care than curative care. CHPS are expected to place more emphases on preventive care such as antenatal care, vaccination, family planning, postnatal Care (PNC). In addition, CHPS seeks to transform primary health care system by shifting the emphasis from the conservative facility-based approach towards a programme of mobile community-based care [31].

In recent times, health facilities are increasingly relying exclusively on Internally Generated Funds (IGF).Our study results showed that the average annual IGF for CHPS and HC were US$2327 and US$15,795 respectively. The national health insurance was the main source of revenue in all the health facilities accounting for over 90% total revenue generated for the period. However, there are reports of delays in NHIS reimbursement and low reimbursement rates for services provided [32].This therefore poses a threat to the financial sustainability and quality of care at the health facilities. To address this situation, more innovative strategies to address delays in reimbursement is urgently needed.

The cost per capita at the CHPS and HC were US$5.14 and US$3.51 respectively. This cost per capita is much lower than the Ghana national average health expenditure per capita of US$58 [33]. Therefore appropriate allocation of resources to the primary health care such as the health centres and CHPS will yield great dividends.

The average cost per OPD attendance at CHPS and HC were US$8.79 and US$5.16 respectively. Our study figures are higher than the figures presented in a previous study that reported average cost per OPD attendance at HC to range between US$2.15 and US$2.68 [8]. A possible explanation could be due to time difference in data collection given that their analysis was based on data collected in 2004/2005(consumer prices inflation in 2004 = 12.6% and 2015 = 17.5%). In addition, location and utilization or population coverage could be reasons for the variations.

In general, budgetary allocations on primary health care in Ghana are not adequate to meet the growing demands for health care. For instance, the Abuja Declaration of 2001 required West African governments to commit at least 15% of their national budgets to health, but in 2015, Ghana’s budget allocation to the health sector was only 9.47% of total spending [34]. However, it is still necessary to make effective use of limited budget.

### Limitations of the study

The use of a sample size of nine primary healthcare facilities may not be large enough to produce results that can be generalized to the entire country. However, considering the homogeneous nature of CHPS compounds and Health Centres in terms of staff, availability of medicines, infrastructure etc.in the country, we don’t expect much variations from the cost estimates obtained in this study. The findings give an indication of the average cost of running a primary healthcare facility in the study region and present an opportunity for similar studies to be conducted in other regions of the country. Similarly, record keeping at the health facilities was poor and could affect the study results. For instance, in some cases, we had to rely on estimations of prices from the market.

We did not carry out time-motion study to determine the staff time on activities as it is being reported to be robust method [29, 35]. However, considering the setting of the health facilities, the method we used in our study for estimation of staff time is appropriate and this methods has been used in other studies to estimate staff time on activities at the primary health care level [29, 36, 37].

Building costs, although important cost component was not included in the cost calculation because we could not obtain reliable cost of building the health facilities. The health facilities were built either by the district assembly or JICA and there were no records available on building costs. Also some of the health facilities had solar panels, but because they were donated to the health facilities and there were no cost records on them, they were not included in the cost analysis.

The cost provided in this study is therefore an underestimation of the cost of providing care at the primary health care facilities given that some cost components like building costs, direct out-of-pocket patient costs and opportunity costs of seeking care at the health facilities were not included in the cost estimations.

## Conclusions

The study findings have added to limited knowledge of cost of providing health care at the health centres and CHPS. The average annual cost of delivering primary health services through CHPS and HCs is US$10,923 and US$44,638 respectively and personnel cost accounts for the major cost. The findings of this study can be useful in cost-effectiveness analysis in related area. Also the government should be guided by these findings in their financial planning, national health insurance refinement, decision making and resource allocation in order to improve primary health care in the country. However, more similar studies involving large numbers of primary health facilities in different parts of the country are needed to assess the cost of providing primary health care.

## Additional file

Additional file 1: Questionnaire for health facility data collection. (DOC 475 kb)

## Abbreviations

ANC: Antenatal Care
CHPS: Community-based Health Planning and Services
DBI: Daffiama Bussie Issa
GHS: Ghana Health Service
HC: Health Centres
IGF: Internally Generated Funds
MOH: Ministry of Health
NHIS: National Health Insurance Scheme
OOP: Out of Pocket
OPD: Outpatient Department
PHC: Primary health care
PNC: Postnatal Care
UHC: Universal Health Coverage
UWR: Upper West Region
