# Supplementary material for: Cost of delivering health care services at primary health facilities in Ghana
Source: BMC Health Serv Res. 2017 Nov 17;17:742. doi: 10.1186/s12913-017-2676-3 (PMC5693519; doi:10.1186/s12913-017-2676-3)
Supplement: Additional file 1: — Questionnaire for health facility data collection. (DOC 475 kb) [file 12913_2017_2676_MOESM1_ESM.doc]

**QUESTIONARE FOR HEALTH FACILITY DATA COLLECTION**

**SECTION A:**

**Table S1: Background**

| No. | Name of district |  |
| --- | --- | --- |
|  | District capital |  |
|  | Sub-District |  |
|  | Name of Health facility |  |
|  | Name of In-charge |  |
|  | Contact number of in-charge |  |
|  | Date of interview |  |

**SECTION B**

Table S2: Health utilization in 2015

| **Number** | **2015** |
| --- | --- |
| OPD(all cases) |  |
| OPD(under fives only) |  |
| Population covered |  |
| Inpatients (if any) |  |

**Table S3: Cases recorded**

| No. | Type of case/illness | Number |
| --- | --- | --- |
|  |  |  |
|  |  |  |
|  |  |  |
|  |  |  |
|  |  |  |
|  |  |  |
|  |  |  |
|  |  |  |
|  |  |  |

**SECTION C**

Table S4: Cash revenue made in 2015

| **Source** | **Amount** |
| --- | --- |
| IGF(user fees/OOP) |  |
| IGF(NHI claims) |  |
|  |  |
|  |  |
|  |  |

**SECTION D**

**Cost of running a health facility (reference year is 2015-january to December)**

1. **PERSONNEL COST**

Table S5**:** Personnel Cost

| **Name of staff** | **Qualification** | **Gross monthly salary** | **Number of months worked** | **Gross salary for the year** | **Complements of earnings (e.g. housing allowances, overtime, transport allowances, uniform, incentives etc.).** | **Total**  **( staff cost for all activities)** |
| --- | --- | --- | --- | --- | --- | --- |
|  |  |  |  |  |  |  |
|  |  |  |  |  |  |  |
|  |  |  |  |  |  |  |
|  |  |  |  |  |  |  |
|  |  |  |  |  |  |  |

**Table S6: Staff time allocation on activities**

| No. | Name of staff | Percentage of time on curative services/ disease treatment: general consultation, nursing, delivery, laboratory, and dispensing drugs | Percentage of time on preventive services: e.g. antenatal, vaccination, family planning, post-natal, and prevention health/health promotion | Percentage of time on other services such guarding etc | Total (100%) |
| --- | --- | --- | --- | --- | --- |
| 1 |  |  |  |  |  |
| 2 |  |  |  |  |  |
| 3 |  |  |  |  |  |
| 4 |  |  |  |  |  |
| 5 |  |  |  |  |  |

**Training cost**

Q1: Are there staff who received training in the last 3 years?

1 = Yes 2 = No

Q2: If yes, how many staff? ………………………………………

**Table S7: Type of training by staff**

| **Name of staff** | **Qualification before the training** | **Type of training**  1= national  2= abroad | **Start year of training** | **Duration of the training** | **Qualification obtained or expected** |
| --- | --- | --- | --- | --- | --- |
|  |  |  |  |  |  |
|  |  |  |  |  |  |
|  |  |  |  |  |  |
|  |  |  |  |  |  |
|  |  |  |  |  |  |

**Table S8: Training cost by staff**

| **Name of staff** | **Transportation cost** | **Living** | **Training fees** | **Salary** | **Bonus** | **Others** |
| --- | --- | --- | --- | --- | --- | --- |
|  |  |  |  |  |  |  |
|  |  |  |  |  |  |  |
|  |  |  |  |  |  |  |
|  |  |  |  |  |  |  |
|  |  |  |  |  |  |  |
|  |  |  |  |  |  |  |
|  |  |  |  |  |  |  |
|  |  |  |  |  |  |  |

1. **BUILDING COST**

Q1: In which year was this health facility built?..............…

Q2: Who constructed the health facility ………..

Q3:What is the construction cost? (local currency)…………………

Q4: How many rooms are in the health facility

**Table S9: Activities carried out in each room**

| **Rooms** | **Activities** |
| --- | --- |
| 1 |  |
| 2 |  |
| 3 |  |
| 4 |  |
| 5 |  |
| 6 |  |

1. **EQUIPMENT (Functioning equipment and furniture)**

Table S10: General equipment

| **Equipment** | **Type/brand** | **Number** | **Unit cost** | **Total** |
| --- | --- | --- | --- | --- |
| Solar panel |  |  |  |  |
| Borehole |  |  |  |  |
| Generator |  |  |  |  |
| Pumping machine |  |  |  |  |
| Water tanks |  |  |  |  |
|  |  |  |  |  |
|  |  |  |  |  |
|  |  |  |  |  |

**Table S11: Equipment per room**

|  | Rooms | | | | | | | | | |  |  |  |
| --- | --- | --- | --- | --- | --- | --- | --- | --- | --- | --- | --- | --- | --- |
| **Equipment** | 1 | 2 | 3 | 4 | 5 | 6 | 7 | 8 | 9 | 10 | Total number | Unit cost | Total cost |
| Bed side locker |  |  |  |  |  |  |  |  |  |  |  |  |  |
| Cannula |  |  |  |  |  |  |  |  |  |  |  |  |  |
| Card Box |  |  |  |  |  |  |  |  |  |  |  |  |  |
| Ceiling Fan |  |  |  |  |  |  |  |  |  |  |  |  |  |
| Clinical thermometer |  |  |  |  |  |  |  |  |  |  |  |  |  |
| Clock /watch |  |  |  |  |  |  |  |  |  |  |  |  |  |
| Cold Box |  |  |  |  |  |  |  |  |  |  |  |  |  |
| Computer |  |  |  |  |  |  |  |  |  |  |  |  |  |
| Cupboard |  |  |  |  |  |  |  |  |  |  |  |  |  |
| Delivery bed |  |  |  |  |  |  |  |  |  |  |  |  |  |
| Dispensary Cabinet |  |  |  |  |  |  |  |  |  |  |  |  |  |
| Dissecting Forceps |  |  |  |  |  |  |  |  |  |  |  |  |  |
| Dressing Forceps |  |  |  |  |  |  |  |  |  |  |  |  |  |
| Drip stand |  |  |  |  |  |  |  |  |  |  |  |  |  |
| Dustbin |  |  |  |  |  |  |  |  |  |  |  |  |  |
| Examination bed |  |  |  |  |  |  |  |  |  |  |  |  |  |
| FAS count |  |  |  |  |  |  |  |  |  |  |  |  |  |
| Fetoscope |  |  |  |  |  |  |  |  |  |  |  |  |  |
| Gallipot |  |  |  |  |  |  |  |  |  |  |  |  |  |
| Given Set |  |  |  |  |  |  |  |  |  |  |  |  |  |
| Haemoglobin analyzer |  |  |  |  |  |  |  |  |  |  |  |  |  |
| Hand washing basin |  |  |  |  |  |  |  |  |  |  |  |  |  |
| Hemoglobin Meter |  |  |  |  |  |  |  |  |  |  |  |  |  |
| ICE chest |  |  |  |  |  |  |  |  |  |  |  |  |  |
| Kidney Dish |  |  |  |  |  |  |  |  |  |  |  |  |  |
| Large medicine cupboard |  |  |  |  |  |  |  |  |  |  |  |  |  |
| Large wooden benches |  |  |  |  |  |  |  |  |  |  |  |  |  |
| Laundry busket |  |  |  |  |  |  |  |  |  |  |  |  |  |
| Mattress |  |  |  |  |  |  |  |  |  |  |  |  |  |
| Medicine cabinet |  |  |  |  |  |  |  |  |  |  |  |  |  |
| Medicine trolley |  |  |  |  |  |  |  |  |  |  |  |  |  |
| Metal Bucket |  |  |  |  |  |  |  |  |  |  |  |  |  |
| Metal chair |  |  |  |  |  |  |  |  |  |  |  |  |  |
| Metal file cabinet |  |  |  |  |  |  |  |  |  |  |  |  |  |
| Metal Table |  |  |  |  |  |  |  |  |  |  |  |  |  |
| Microscope |  |  |  |  |  |  |  |  |  |  |  |  |  |
| Needle half circle 16 |  |  |  |  |  |  |  |  |  |  |  |  |  |
| Octoclave |  |  |  |  |  |  |  |  |  |  |  |  |  |
| Photocopier |  |  |  |  |  |  |  |  |  |  |  |  |  |
| Plasmar seperator |  |  |  |  |  |  |  |  |  |  |  |  |  |
| Plastic bucket |  |  |  |  |  |  |  |  |  |  |  |  |  |
| Plastic Chair |  |  |  |  |  |  |  |  |  |  |  |  |  |
| Plastic Table |  |  |  |  |  |  |  |  |  |  |  |  |  |
| Printer |  |  |  |  |  |  |  |  |  |  |  |  |  |
| Radio |  |  |  |  |  |  |  |  |  |  |  |  |  |
| Refrigerator |  |  |  |  |  |  |  |  |  |  |  |  |  |
| Safe |  |  |  |  |  |  |  |  |  |  |  |  |  |
| Sample taken bench |  |  |  |  |  |  |  |  |  |  |  |  |  |
| Scaler |  |  |  |  |  |  |  |  |  |  |  |  |  |
| Scalp vein |  |  |  |  |  |  |  |  |  |  |  |  |  |
| Screen |  |  |  |  |  |  |  |  |  |  |  |  |  |
| Sink |  |  |  |  |  |  |  |  |  |  |  |  |  |
| Small cupboard |  |  |  |  |  |  |  |  |  |  |  |  |  |
| Small wooden benches |  |  |  |  |  |  |  |  |  |  |  |  |  |
| Spectrophotometer |  |  |  |  |  |  |  |  |  |  |  |  |  |
| Sphygmomanometer |  |  |  |  |  |  |  |  |  |  |  |  |  |
| Stabilizer |  |  |  |  |  |  |  |  |  |  |  |  |  |
| Steriliser /stove 4 burner |  |  |  |  |  |  |  |  |  |  |  |  |  |
| Stethoscope |  |  |  |  |  |  |  |  |  |  |  |  |  |
| Stool |  |  |  |  |  |  |  |  |  |  |  |  |  |
| Stretcher |  |  |  |  |  |  |  |  |  |  |  |  |  |
| Suction machine |  |  |  |  |  |  |  |  |  |  |  |  |  |
| Swivel Chair |  |  |  |  |  |  |  |  |  |  |  |  |  |
| Television |  |  |  |  |  |  |  |  |  |  |  |  |  |
| Theater trolley |  |  |  |  |  |  |  |  |  |  |  |  |  |
| Vofex |  |  |  |  |  |  |  |  |  |  |  |  |  |
| Walking Aid |  |  |  |  |  |  |  |  |  |  |  |  |  |
| Water bath |  |  |  |  |  |  |  |  |  |  |  |  |  |
| Water dispensor |  |  |  |  |  |  |  |  |  |  |  |  |  |
| Water distiler |  |  |  |  |  |  |  |  |  |  |  |  |  |
| Weighing scale for adults |  |  |  |  |  |  |  |  |  |  |  |  |  |
| Weighing scale for babies |  |  |  |  |  |  |  |  |  |  |  |  |  |
| Wheel chair |  |  |  |  |  |  |  |  |  |  |  |  |  |
| Wooden Cabinet |  |  |  |  |  |  |  |  |  |  |  |  |  |
| Wooden chair |  |  |  |  |  |  |  |  |  |  |  |  |  |
| Wooden Table |  |  |  |  |  |  |  |  |  |  |  |  |  |
|  |  |  |  |  |  |  |  |  |  |  |  |  |  |

1. **TRANSPORTAION COST**

**Table S12: Means of transport**

| **Items** | **Type** | **Number** | **Purpose of means of transport** | **Unit cost** | **Total cost** |
| --- | --- | --- | --- | --- | --- |
| Motor cycles |  |  |  |  |  |
|  |  |  |  |  |  |
|  |  |  |  |  |  |
|  |  |  |  |  |  |
| Four wheel vehicle |  |  |  |  |  |
|  |  |  |  |  |  |
| Three wheel vehicle |  |  |  |  |  |
|  |  |  |  |  |  |
|  |  |  |  |  |  |
| Ambulance |  |  |  |  |  |
| Bicycle |  |  |  |  |  |
|  |  |  |  |  |  |
|  |  |  |  |  |  |
|  |  |  |  |  |  |

1. **DRUGS AND MEDICAL CONSUMABLES**

**Table S13: Drugs and medical consumables**

| **Name Of Drug and consumables** | **Jan** | **Feb** | **march** | **April** | **May** | **June** | **July** | **August** | **Sept** | **Oct** | **Nov** | **Dec** | **Total** | **unit price** |  |
| --- | --- | --- | --- | --- | --- | --- | --- | --- | --- | --- | --- | --- | --- | --- | --- |
| **TABS** |  |  |  |  |  |  |  |  |  |  |  |  |  |  |  |
| Aluminium hydroxide |  |  |  |  |  |  |  |  |  |  |  |  |  |  |  |
| arteunate amodiaquine (adult) |  |  |  |  |  |  |  |  |  |  |  |  |  |  |  |
| Artesonate-amodiaquine(1-5 years) |  |  |  |  |  |  |  |  |  |  |  |  |  |  |  |
| Artesonate-amodiaquine(6-13 years) |  |  |  |  |  |  |  |  |  |  |  |  |  |  |  |
| Artesonate-amodiaquine(0-11 months) |  |  |  |  |  |  |  |  |  |  |  |  |  |  |  |
| Arthemter lumefantrine |  |  |  |  |  |  |  |  |  |  |  |  |  |  |  |
| Ibuprofen-200 |  |  |  |  |  |  |  |  |  |  |  |  |  |  |  |
| Ibuprofen-400 |  |  |  |  |  |  |  |  |  |  |  |  |  |  |  |
| ciprofluxacin |  |  |  |  |  |  |  |  |  |  |  |  |  |  |  |
| co-amoxazole |  |  |  |  |  |  |  |  |  |  |  |  |  |  |  |
| diclofenac |  |  |  |  |  |  |  |  |  |  |  |  |  |  |  |
| diazepam |  |  |  |  |  |  |  |  |  |  |  |  |  |  |  |
| Ferrus sulphate |  |  |  |  |  |  |  |  |  |  |  |  |  |  |  |
| Folic Acid |  |  |  |  |  |  |  |  |  |  |  |  |  |  |  |
| Mebendazole |  |  |  |  |  |  |  |  |  |  |  |  |  |  |  |
| paracetamol |  |  |  |  |  |  |  |  |  |  |  |  |  |  |  |
| promethazine |  |  |  |  |  |  |  |  |  |  |  |  |  |  |  |
|  |  |  |  |  |  |  |  |  |  |  |  |  |  |  |  |
| **CAPSULES** |  |  |  |  |  |  |  |  |  |  |  |  |  |  |  |
| Amoxycillin-250 |  |  |  |  |  |  |  |  |  |  |  |  |  |  |  |
| Amoxycillin-500 |  |  |  |  |  |  |  |  |  |  |  |  |  |  |  |
| Chloramphenicol |  |  |  |  |  |  |  |  |  |  |  |  |  |  |  |
| Doxycilline |  |  |  |  |  |  |  |  |  |  |  |  |  |  |  |
| Flucloxacine |  |  |  |  |  |  |  |  |  |  |  |  |  |  |  |
| Tetrecycline |  |  |  |  |  |  |  |  |  |  |  |  |  |  |  |
| Multivitamin |  |  |  |  |  |  |  |  |  |  |  |  |  |  |  |
| iron III polymatose |  |  |  |  |  |  |  |  |  |  |  |  |  |  |  |
|  |  |  |  |  |  |  |  |  |  |  |  |  |  |  |  |
| **INJECTIONS** |  |  |  |  |  |  |  |  |  |  |  |  |  |  |  |
| diclofenac |  |  |  |  |  |  |  |  |  |  |  |  |  |  |  |
| diaxepam |  |  |  |  |  |  |  |  |  |  |  |  |  |  |  |
| Gentamycine |  |  |  |  |  |  |  |  |  |  |  |  |  |  |  |
| promethazine |  |  |  |  |  |  |  |  |  |  |  |  |  |  |  |
| Oxytocin |  |  |  |  |  |  |  |  |  |  |  |  |  |  |  |
| Buscopan |  |  |  |  |  |  |  |  |  |  |  |  |  |  |  |
|  |  |  |  |  |  |  |  |  |  |  |  |  |  |  |  |
| **SUSPENSIONS** |  |  |  |  |  |  |  |  |  |  |  |  |  |  |  |
| Amoxycillin |  |  |  |  |  |  |  |  |  |  |  |  |  |  |  |
| Co-trimoxazole |  |  |  |  |  |  |  |  |  |  |  |  |  |  |  |
| Flucloxacillin |  |  |  |  |  |  |  |  |  |  |  |  |  |  |  |
| Metronidazole |  |  |  |  |  |  |  |  |  |  |  |  |  |  |  |
| Erythromycin |  |  |  |  |  |  |  |  |  |  |  |  |  |  |  |
|  |  |  |  |  |  |  |  |  |  |  |  |  |  |  |  |
| **SYRUPS** |  |  |  |  |  |  |  |  |  |  |  |  |  |  |  |
| paracetamol |  |  |  |  |  |  |  |  |  |  |  |  |  |  |  |
| Multivitamin |  |  |  |  |  |  |  |  |  |  |  |  |  |  |  |
| promethazine |  |  |  |  |  |  |  |  |  |  |  |  |  |  |  |
|  |  |  |  |  |  |  |  |  |  |  |  |  |  |  |  |
| **OINTMENT** |  |  |  |  |  |  |  |  |  |  |  |  |  |  |  |
| diclofenac gel |  |  |  |  |  |  |  |  |  |  |  |  |  |  |  |
| White field ointment |  |  |  |  |  |  |  |  |  |  |  |  |  |  |  |
| Tetrecycline |  |  |  |  |  |  |  |  |  |  |  |  |  |  |  |
| Clotrimozola cream |  |  |  |  |  |  |  |  |  |  |  |  |  |  |  |
| Cotrimoxazole passeries |  |  |  |  |  |  |  |  |  |  |  |  |  |  |  |
| Kalamin lotion |  |  |  |  |  |  |  |  |  |  |  |  |  |  |  |
| Chloramphenicol ear drop |  |  |  |  |  |  |  |  |  |  |  |  |  |  |  |
| Chloramphenicol eye drop |  |  |  |  |  |  |  |  |  |  |  |  |  |  |  |
| paracetamol supportory |  |  |  |  |  |  |  |  |  |  |  |  |  |  |  |
| cotton |  |  |  |  |  |  |  |  |  |  |  |  |  |  |  |
| Gauze |  |  |  |  |  |  |  |  |  |  |  |  |  |  |  |
| Spirit |  |  |  |  |  |  |  |  |  |  |  |  |  |  |  |
| liquid soap |  |  |  |  |  |  |  |  |  |  |  |  |  |  |  |
|  |  |  |  |  |  |  |  |  |  |  |  |  |  |  |  |
|  |  |  |  |  |  |  |  |  |  |  |  |  |  |  |  |
|  |  |  |  |  |  |  |  |  |  |  |  |  |  |  |  |
| **Comsumaerbles consumed** |  |  |  |  |  |  |  |  |  |  |  |  |  |  |  |
| Cotton |  |  |  |  |  |  |  |  |  |  |  |  |  |  |  |
| Bandage |  |  |  |  |  |  |  |  |  |  |  |  |  |  |  |
| Dispensing evelop |  |  |  |  |  |  |  |  |  |  |  |  |  |  |  |
| Paster |  |  |  |  |  |  |  |  |  |  |  |  |  |  |  |
| Gauze B. (rolls) |  |  |  |  |  |  |  |  |  |  |  |  |  |  |  |
| Surgical gloves (Box) |  |  |  |  |  |  |  |  |  |  |  |  |  |  |  |
| Plaster (rolls) |  |  |  |  |  |  |  |  |  |  |  |  |  |  |  |
| Liquid soap (Galon) |  |  |  |  |  |  |  |  |  |  |  |  |  |  |  |
| Parazone(galon) |  |  |  |  |  |  |  |  |  |  |  |  |  |  |  |
|  |  |  |  |  |  |  |  |  |  |  |  |  |  |  |  |
| RDTs |  |  |  |  |  |  |  |  |  |  |  |  |  |  |  |
| HIV test kits |  |  |  |  |  |  |  |  |  |  |  |  |  |  |  |
| Syphilis teat kits |  |  |  |  |  |  |  |  |  |  |  |  |  |  |  |

1. **ADMINISTRATIVE COST**

**Table S14:** Administrative costs

| **Items** | **Amount** |
| --- | --- |
| Electricity(VRA) |  |
| Water |  |
| Telephone |  |
| Cleaning products |  |
| Repairs, plumbing, roofs etc. |  |
| Spare parts |  |
| Servicing fees |  |
| Fuel |  |
| Lubricants |  |
| Post office |  |
| Printing and photocopying |  |
| **others** |  |
|  |  |
|  |  |
|  |  |
|  |  |
|  |  |

**Table S15: Stationery**

| Name of stationery | Quantity consumed | Unit price | Total | Total value |
| --- | --- | --- | --- | --- |
| Folders |  |  |  |  |
| Files arch |  |  |  |  |
| Flat files |  |  |  |  |
| Stapler |  |  |  |  |
| Staple pins |  |  |  |  |
| A 4 paper |  |  |  |  |
| A3 paper |  |  |  |  |
| Envelopes (A4) |  |  |  |  |
| Envelopes (A3) |  |  |  |  |
| Pencils(2B) |  |  |  |  |
| Pencils (HB) |  |  |  |  |
| Pens (Bic) |  |  |  |  |
| Record card |  |  |  |  |
| Notebooks |  |  |  |  |
| Maternal health book |  |  |  |  |
| Child health book |  |  |  |  |
| Others (specify) |  |  |  |  |
|  |  |  |  |  |
|  |  |  |  |  |
|  |  |  |  |  |
|  |  |  |  |  |
|  |  |  |  |  |
|  |  |  |  |  |
|  |  |  |  |  |
